# Retraction: JQ1 suppresses tumor growth via PTEN/PI3K/AKT pathway in endometrial cancer

**DOI:** 10.18632/oncotarget.28867

**Published:** 2026-04-24

**Authors:** Haifeng Qiu, Jing Li, Leslie H. Clark, Amanda L. Jackson, Lu Zhang, Hui Guo, Joshua E. Kilgore, Paola A. Gehrig, Chunxiao Zhou, Victoria L. Bae-Jump

**Affiliations:** ^1^Department of Obstetrics and Gynecology, the First Affiliated Hospital of Zhengzhou University, Zhengzhou, China; ^2^Division of Gynecological Oncology, University of North Carolina at Chapel Hill, Chapel Hill, NC, USA; ^3^Department of Oncology, the First Affiliated Hospital of Zhengzhou University, Zhengzhou, China; ^4^Department of Gynecologic Oncology, Shandong Cancer Hospital and Institute, Jinan, Chin; ^5^Lineberger Cancer Center, University of North Carolina at Chapel Hill, Chapel Hill, NC, USA

**This article has been retracted:** The University of North Carolina at Chapel Hill conducted a research misconduct proceeding that involved this article and concluded that research misconduct occurred, specifically the falsification with reuse of western blot bands from different experimental conditions in Figures 1E and 3D; and falsification with reuse of immunostained images from different experimental conditions in Figures 2C and 5G. Therefore, the results and conclusions of this publication are no longer reliable. The authors requested the article retraction. Consequently, the Editorial decision has been made to retract this article. All authors agree with this decision, with the exception of Dr. Haifeng Qiu and Dr. Jing Li, who did not respond.

Original article: Oncotarget. 2016; 7:66809–66821. 66809-66821. https://doi.org/10.18632/oncotarget.11631

